# Reinforcement of a minor alternative splicing event in *MYO7A* due to a missense mutation results in a mild form of retinopathy and deafness

**Published:** 2010-09-30

**Authors:** Imen Ben Rebeh, Madeleine Morinière, Leila Ayadi, Zeineb Benzina, Ilhem Charfedine, Jamel Feki, Hammadi Ayadi, Abdelmonem Ghorbel, Faouzi Baklouti, Saber Masmoudi

**Affiliations:** 1 Unité Cibles pour le Diagnostic et la Thérapie, Centre de Biotechnologie de Sfax, Tunisie; 2 mRNA Metabolism in Normal and Pathological Cells, CGMC, CNRS, Université Lyon 1, Villeurbanne, France; 3 Service d’Ophtalmologie, C.H.U.H. Bourguiba de Sfax, Tunisie; 4 Service d'O.R.L., C.H.U. H. Bourguiba de Sfax, Tunisie

## Abstract

**Purpose:**

Recessive mutations of the myosin VIIA (*MYO7A)* gene are reported to be responsible for both a deaf–blindness syndrome (Usher type 1B [USH1B] and atypical Usher syndrome) and nonsyndromic hearing loss (HL; Deafness, Neurosensory, Autosomal Recessive 2 [DFNB2]). The existence of DFNB2 is controversial, and often there is no relationship between the type and location of the *MYO7A* mutations corresponding to the USH1B and DFNB2 phenotype. We investigated the molecular determinant of a mild form of retinopathy in association with a subtle splicing modulation of *MYO7A* mRNA.

**Methods:**

Affected members underwent detailed audiologic and ocular characterization. DNA samples from family members were genotyped with polymorphic microsatellite markers. Sequencing of *MYO7A* was performed. Endogenous lymphoid RNA analysis and a splicing minigene assay were used to study the effect of the c.1935G>A mutation.

**Results:**

Funduscopy showed mild retinitis pigmentosa in adults with HL. Microsatellite analysis showed linkage to markers in the region on chromosome 11q13.5. Sequencing of *MYO7A* revealed a mutation in the last nucleotide of exon 16 (c.1935G>A), which corresponds to a substitution of a methionine to an isoleucine residue at amino acid 645 of the myosin VIIA. However, structural prediction of the molecular model of myosin VIIA shows that this amino acid replacement induces only minor structural changes in the immediate environment of the mutation and thus does not alter the overall native structure. We found that, although predominantly included in mature mRNA, exon 16 is in fact alternatively spliced in control cells and that the mutation at the very last position is associated with a switch toward a predominant exclusion of that exon. This observation was further supported using a splicing minigene transfection assay; the c.1935G>A mutation was found to trigger a partial impairment of the adjacent donor splice site, suggesting that the unique change at the last position of the exon is responsible for the enhanced exon exclusion in this family.

**Conclusions:**

This study shows how an exonic mutation that weakens the 5′ splice site enhances a minor alternative splicing without abolishing a complete exclusion of the exon and therefore causes a less severe retinitis pigmentosa than the USH1B-associated alleles. It would be interesting to examine a possible correlation between intrafamilial phenotypic variability and the subtle variation in exon 16 inclusion, probably related to genetic background specificities.

## Introduction

Myosin VIIA, an unconventional myosin, is a member of a large superfamily of actin-associated molecular motors. It is composed of a structurally conserved head, neck, and tail regions. The latter binds actin and hydrolyzes ATP to produce force and movement. Myosin VIIA physiologic function is best studied in the sensory hair cells of the inner ear and the retina. In the inner ear, myosin VIIA is required for hair bundle morphogenesis and mechanotransduction [1,2]. Within the retina, myosin VIIA localizes to the cilium of the photoreceptors, to the apical region of retinal pigment epithelium (RPE) cells, and to melanosome within RPE cells [3-5]. In accordance with its expression pattern in retina, myosin VIIA regulates opsin and melanosome transports and the phagocytosis of shed outer segments by RPEs [6,7]. Myosin VIIA has been implicated in recessively inherited Usher syndrome type 1B (USH1B) [8], atypical Usher syndrome (USH3) [9], nonsyndromic recessive (Deafness, Neurosensory, Autosomal Recessive 2 [DFNB2]) [10], and dominant (DFNA11) [11] hearing loss (HL). USH1B is clinically characterized by prelingual severe to profound HL, prepubertal progressive retinitis pigmentosa (RP), and vestibular areflexia. A progressive HL, variable vestibular problems, and RP are characteristic of USH3. Although the existence of myosin VIIA (*MYO7A)* recessive mutations that are associated with nonsyndromic HL phenotype is still controversial, there is evidence of variability in the clinical onset and diagnosis of RP among patients with *MYO7A* mutations. In the large Tunisian family used to define the DFNB2 locus, funduscopy revealed mild RP in five out of 25 affected persons with HL [12]. In the Pakistani DFNB2 family, one deaf patient (41 years old) had slightly subnormal rod and cone responses and a suboptimal quality electroretinogram (ERG).

Over 130 mutations in *MYO7A* have been identified and are listed in the Human Gene Mutation Database, most leading to a diagnosis of USH1B. Mutations in *MYO7A* were reported in five families with nonsyndromic recessive HL. It was hypothesized that DFNB2 mutations cause a less severe phenotype than the USH1B-associated alleles because the resulting protein retains some degree of normal function, at least in retina. This hypothesis was confirmed only in the DFNB2 Pakistani family. Riazuddin et al. [13] showed that green fluorescent protein (GFP)-tagged form of myosin VIIa containing deletion p.E1716del localizes properly to stereocilia in transfected mouse inner ear hair cells, similarly to the wild-type protein, which argues for the residual functional activity of the altered protein.

Using genetic linkage and sequencing analyses, we identified a missense mutation (c.1935G>A) in a Tunisian family segregating nonsyndromic HL. Funduscopy showed that RP is mild in adult patients. The mutation is located at the last nucleotide of *MYO7A* exon 16. The altered mRNA displayed a predominant exclusion of exon 16. A functional analysis using splicing minigene transfection assay further supported the effect of the mutation on exon exclusion.

## Methods

### Family and clinical evaluation

**Figure 1:**
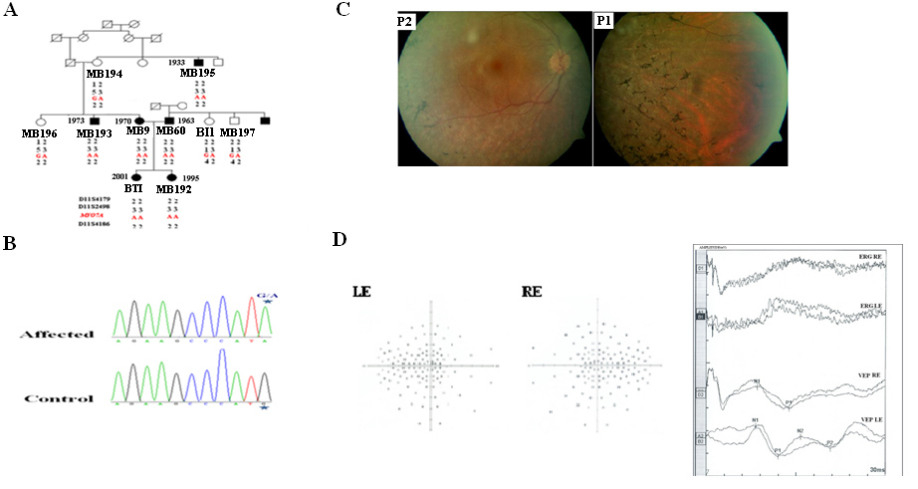
Pedigree and analysis of a Tunisian family segregating autosomal recessive hearing loss with variability in the diagnosis of retinitis pigmentosa. **A**: Pedigree of a Tunisian family and haplotypes of polymorphic markers in the *MYO7A* region are shown. In pedigree, the square symbol indicates male, the circle symbol denotes female and black symbol represents affected individuals. **B**: DNA chromatogram from a control individual (bottom) and patient MB9 (top) are shown. Mutation consists on a homozygous mutation (G-A) at nucleotide 1935 located in exon 16, resulting in a Met to Ile substitution at amino acid 645 of myosin VIIA. Asterisk indicates the position of the homozygous mutation. **C**: Fundus photographs in 40-year-old (P1: MB9) and 47-year-old (P2: MB60) patients showed variability in the severity of retinitis pigmentosa (RP). MB9 had a mild peripheral RP, while MB60 had only some pigments. **D**: Visual field test results obtained on the right (RE) and the left (LE) eye of patient MB9 at the age of 40 years are shown. A series of random lights of different intensities are flashed in the peripheral field of vision of the patient. When the patient perceives the computer generated light suddenly appearing in his or her field of view, the patient presses a button to indicate a response. In the picture of the visual field a lighter gray spot is assigned. If the patient is unable to see the light in an appropriate portion of the field of view, then we see on the computer a darker gray spot (Dot don’t see) indicating vision loss. Visual field loss was severe in this patient. In fact, the nasal and temporal fields were not preserved and only the central field was maintained. Ganzfeld electroretinogram (ERG) and visual-evoked potentials (VEP) of RE and LE of patient MB9 are shown. The ERG and the VEP test the function of the visual pathway from the retina (ERG) to the occipital cortex (VEP). These tests were conducted by placing a standard ERG device attached to the skin 2 mm above the orbit. VEPs were recorded simultaneously from an electrode attached to the occipital scalp 2 mm above the region on the midsagittal plane. An electrode placed on the forehead provided a ground. The results can be directly related to the part of a visual field that might be defective. This is based on the anatomic relationship of the retinal images and the visual field. After dark adaptation for 30 min, the doctor places anesthetic drops in the patient's eye and places a contact lens on the surface of the eye. Once the contact lens is in place, a series of blue, red, and white lights is shown to the patient. The VEP is an evoked electrophysiological potential that can be extracted, using signal averaging, from the electroencephalographic activity recorded at the scalp. Both the ERG and VEP were differentially amplified band pass filtered (0, 1, 30 Hz), recorded over 300 ms epochs, and signal averaged. The visual evoked potential to flash stimulation consists of a series of negative and positive waves. The earliest detectable response has a peak latency of approximately 30 ms post stimulus. For the flash VEP, the most robust components are the negative peak N2 and positive peak P2 peaks. Measurements of the P2 amplitude should be made from the positive P2 peak at around 207.3 ms. The ERG recorded in MB9 showed an absence of responses. While the VEP showed a normal response in both eyes. These traces confirm the evidence of a significant bilateral global retinal degeneration. Only cone flicker responses of less than 15% of the normal mean were recordable under photopic conditions, while all other responses were below noise level, a typical finding for patients with RP.

Two deaf individuals from a Tunisian family were enrolled through a deaf school. During a home visit, we ascertained additional four deaf individuals (Figure 1). Informed consent was obtained from patients and control individuals in accordance with the ethics committee of the University Hospital of Sfax. Pure tone audiometry was performed. Affected members underwent evaluation for balance using caloric testing and ophthalmological investigation, including fundus ophthalmoscopy. Clinical history and physical examinations of family members ruled out the implication of environmental factors in the etiology of HL and RP. Ten ml of blood samples have been taken from the vein in the antecubital fossa from ten family members. Immediately, tubes were inverted about 5 times and labeled with the subject identification code. Samples were refrigerated to 4 °C for no more than 4 h. Genomic DNA was extracted from whole blood following a standard phenol-chloroform method. Briefly, lymphocytes were incubated in a solution of Sodium dodecyl sulfate detergent and proteinase K. The DNA solution is first extracted with a phenol/chloroform/isoamyl alcohol mixture then precipitated with 100% ethanol. The DNA is pelleted after the precipitation step, washed with 70% ethanol, and resuspended in Tris-EDTA buffer.

### Microsatellites genotyping and mutation analysis

For each gene and locus responsible for USH, at least two microsatellite markers were selected on the basis of their map position and heterozygosity coefficient. Fluorescent dye-labeled microsatellite markers were genotyped for all the participating family members. We used the True Allele PCR Premix (Applied Biosystems, Foster City, CA) for PCR reactions according to the manufacturer’s instructions. Briefly, we assembled a 15 ml reaction containing 9 ml true allele PCR premix, 10 pmole of a mix of forward and reverse primers, and 50 ng of genomic DNA. PCR conditions were as follows: 11 min at 94 °C followed by 35 cycles, each consisting of 15 s at 94 °C, 15 s at 55 °C, and 30 s at 72 °C, then a final elongation at 72 °C. Fluorescently labeled alleles were analyzed on an ABI PRISM 3100-Avant automated genetic analyzer (Applied Biosystems). Genotypes were determined using the GenScan™ and GenoTyper™ software (Applied Biosystems).

One affected subject was investigated for the presence of a mutation in *MYO7A*. Amplified products of all coding exons and exon–intron junctions were directly sequenced using an ABI 3100-Avant automated DNA sequencer and Big Dye Terminator Sequencing V3.1 kit (Applied Biosystems). Screening of the c.1935G>A mutation in the family was performed using PCR-restriction fragment length polymorphism. PCR amplification of a 349 bp DNA fragment, including exon 16 of the *MYO7A* gene, was performed with the primers *MYO7A16F* (5′ACC TCC CCT CCC GCT TCC T3′) and *MYO7A16*R (5′-GCC CCC CAT TCC CCA AAG G-3′). PCR products were digested with 10 U of NcoI restriction endonuclease (New England Biolabs, Ipswich, MA) for 1 h at 37 °C. Digestion of the wild-type allele yielded two fragments of 223 bp and 126 bp, while the mutation abolished the *N*coI site.

### Molecular modeling and prediction of the splice consensus score

The MYO7A sequence (accession number Q13402 in the UniProtKB/Swiss-Protdatabase) was submitted to a Basic Local Alignment Search Tool (BLAST) [14] search against the structures in the protein data bank (PDB) to find suitable templates for homology modeling. The myosin II motor domain from *Dictyostelium discoideum* was found to be the best homolog, with 42% amino acid sequence identity (PDB 1d0x, 2.0 Å resolution [15]) for the query search. The alignment of the myosin VIIa sequence against the template sequence was used as input to the MODELER program [16], together with the atomic coordinates of the latter. Fifty homology models were built by MODELER. The model having the lowest value of the MODELER objective function was selected and was improved by energy minimization. The stereochemical quality of the selected model was evaluated using the PROCHECK program [17]. Swiss-Pdb Viewer [18] was used for structural analyses and to generate the p.M645I mutant model. ConSeq [19] and GetArea version 1.1 using a sphere probe radius of 1.4 Å [20] were used to analyze solvent accessibility surface.

To assess the likelihood of creating or eliminating splice sites of the c.1935G>A mutation, splice site scores were predicted by the Neural Network Based Program and the Splice View program.

### Minigene constructs

To validate in silico prediction of the impact of the c.1935G>A mutation on splicing, wild-type and corresponding mutated genomic fragments were subcloned into expression vectors p(13,17)/cytomegalovirus (CMV) and transfected into HeLa cells. Amplification of *MYO7A* exon 16 and surrounding intronic sequences was performed using a forward primer (5′-**TGA GGT AA**C CAA GTC CGA TTA CTC CTT-3′) and a reverse primer (5′-**GTG CTA GC**G GGC ATC TGC AAG CAT TAC T-3′). Sequence mismatches were introduced to create BstEII and NheI restriction sites (bold letters). These primers led to an 892-bp PCR product. PCR conditions were as follows: 5 min at 94 °C followed by 40 cycles, each consisting of 30 s at 94 °C, 30 s at 66 °C, and 50 s at 72 °C, then a final elongation at 72 °C for 10 min. The PCR products were digested with BstEII and NheI restriction endonucleases and inserted at the BstEII/NheI sites of the splicing cassette p(13,17)/CMV that was designed to contain the two adjacent constitutive exons 13 and 17 of the human 4.1R gene with their downstream and upstream flanking intron sequences, respectively [21]. The constructs were further sequenced to ascertain the absence of additional sequence changes.

### Cell culture and transfection

HeLa cells were cultured in Dulbecco’s modified Eagle’s medium (Invitrogen, Karlsruhe, Germany)with 10% fetal calf serum in four well plates. Cells were transfected with 2 μg of wild-type or mutated minigene constructs, using FuGENE 6 Transfection Reagent (Roche Diagnostics Ltd, Lewes, UK), according to the manufacturer’s procedures. Briefly, a mixture of 95 μl of serum-free medium and 3 µl of FuGENE® 6 was vortexed and incubated for 5 min at room temperature, then, we added 2 μg of plasmid DNA into the tube. After incubation at room temperature, we added the transfection reagent to the cells in a drop-wise manner. Stably transfected cells were selected for 10–12 days in the same medium containing 600–800 μg Geneticin G418/ml (Invitrogen).

### RNA extraction and reverse transcriptase-PCR analysis

HeLa cells were washed twice with Ca^2+^/Mg^2+^-free phosphate-buffered saline (PBS; 1X), collected by trypsinization, and centrifuged. Isolation of total RNA was performed using TRIZOL reagents (Invitrogen), according to the manufacturer’s protocol. RNA was reverse transcribed, and cDNAs were used as templates for PCR amplification using the forward and reverse primers within the cassette’s upstream (UE, 4.1R exon 13) and downstream (DE, 4.1R exon 17) exons, as previously described [21]. Direct sequencing of reverse transcriptase (RT)-PCR products was performed by standard conditions.

The effect of a splice site mutation was also assessed by RT–PCR analysis of lymphoid RNAs obtained from affected and control individuals. Total RNA was isolated from 10 ml of blood samples, using PureLink™ Micro-to-Midi Total RNA Purification System (Invitrogen). Reverse transcription was performed using oligo-dT primers and 200 U of M-MuLV (Moloney Murine Leukemia Virus) Reverse Transcriptase (Fermentas, St. Leon-Rot, Germany). The PCR step was performed using forward primer 5′-AGA CCC AGT TTG GCA TCA AC-3′ (exon 14) and reverse primer 5′-ATT CCT GAG TAC CGC AGC TGG-3′ (exon 17) and was expected to yield a 346-bp fragment.

## Results

### Clinical variability of retinitis pigmentosa within a family segregating hearing loss

An audiometric test performed in patients from the Tunisian family showed severe to profound bilateral sensorineural HL. Deaf individuals who underwent a caloric test had vestibular areflexia. Clinical interviews ruled out any history of a delay in the age of walking. The ophthalmological investigation in this family showed variability in the clinical onset and diagnosis of RP among patients. Individual MB9 (40 years old), her brother (37 years old), and her uncle (77 years old) had similar mild RP (Figure 1). In these individuals, night blindness was reported in the third decade. A visual field test and ERG was performed only in individual MB9. The visual field (Goldmann targets III/4e) was significantly reduced to a 5° concentric field and temporal island fields for both eyes. The nasal and temporal fields were not preserved, and only the central field was maintained (Figure 1). The Ganzfeld-ERG showed an almost normal response flash visual-evoked potential in both eyes and a significant bilateral global retinal degeneration. Only cone flicker responses of less than 15% of the normal mean were recordable under photopic conditions, while all other responses were below noise level, a typical finding for patients with retinitis pigmentosa (Figure 1). Fundus examination of MB9’s non-consanguineous husband (47 years of age) showed only a slight waxy pallor of the optic disc, so the absence of severe retinal degeneration as indicated by thinning and loss of pigment epithelium. Their daughters (15 and 9 years old) did not show any anomaly in funduscopy.

### A point mutation within *MYO7A* revealed by microsatellite genotyping and nucleotide sequencing

In an attempt to identify the altered gene, we first performed a genetic linkage analysis of fluorescent dye-labeled polymorphic microsatellite markers covering all known loci for USH. This analysis revealed evidence for a linkage to *MYO7A* mapping to chromosome 11q13.5 (Figure 1). All affected individuals showed a homozygous haplotype. Direct sequencing of *MYO7A* in one affected individual revealed a G to A transition (c.1935G>A) in the homozygous state. Co-segregation of the transition with HL in the family was confirmed by PCR-restriction fragment length polymorphism. This mutation is located in the last position of exon 16 and would potentially substitute an isoleucine for a methionine at position 645 of the protein (p.M645I).

### Single amino acid substitution would not alter myosin VIIa function

**Figure 2:**
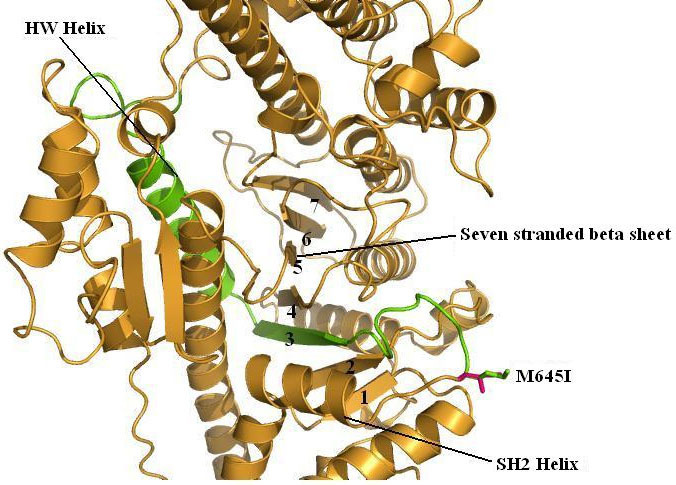
Homology model of human myosin VIIA showing the exon 16-encoded peptide in green in the middle of the motor domain, which is colored in orange. This exon encodes the HW helix [22], the third strand of the central seven-stranded β-sheet, and the associated loops. Wild-type methionine and mutant isoleucine 645 residues before the SH2 helix (in orange) are represented as sticks and highlighted in green and magenta, respectively. Structure visualization was done using Pymol software.

A model covering the myosin motor domain from residue Val2 to Gln752 was built using the crystal structure of the myosin-II heavy chain from *D. discoideum* (PDB 1d0x, 2.0 Å resolution) as a template. The region encoded by exon 16 is located in the middle of this domain, which encodes the HW helix, the third strand of the central seven-stranded β sheet, and two loops (Figure 2). This portion of the protein is highly conserved among myosin VIIA homologs and seems to play an important role in the communication between the actin interface and nucleotide-binding pocket [22]. ConSeq and GetArea results predict that p.Met645, which belongs to a loop between the third strand of the central seven-stranded β sheet and the SH2 helix, is a solvent-exposed residue. The replacement of this solvent-exposed residue with isoleucine does not perturb the native structure. In addition, this mutation is quite distant from the nucleotide and actin-binding sites. Altogether these predictions did not support a major functional impact of the amino acid substitution on protein function. We therefore hypothesized that the genomic mutation must have rather an earlier impact on mRNA metabolism.

### Nucleotide change weakens the 5′ splice site

The c.1935G>A mutation occurs at the last position of exon 16. We used the two splice site prediction programs Neural Network Splice Site Prediction Tool and Splice view to evaluate the strength of the altered splice site. The wild-type sequence resulted in predicted scores of 0.92 and 0.90, while the calculated values of the numerical score for the altered sequence were 0.62 and 0.60, respectively. These data suggest a weakening of the exon 16 donor site caused by the c.1935G>A mutation.

### Single nucleotide mutation enhances exon 16 exclusion

**Figure 3:**
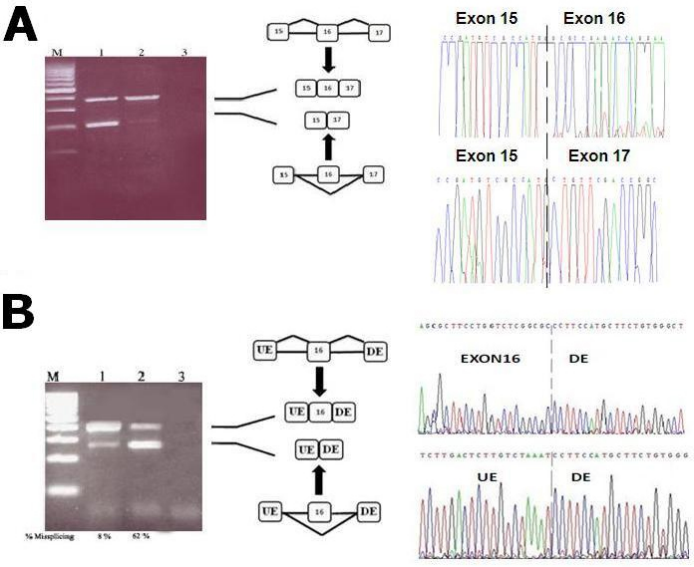
Exon 16 splicing pattern associated with the c.1935G>A mutation. **A**: Analysis of endogenous RNA from lymphoid cells. We amplified a 346-bp fragment from exon 14 to exon 17 of *MYO7A* in a healthy control individual. An additional faint band appeared in the control; it corresponded to an mRNA species missing exon 16 (lane 2). In patient MB9, the splicing pattern showed a predominant exclusion of exon 16 (lane 1). Lane 3 refers to negative control and M indicates size marker. Direct sequencing of these two different transcripts revealed that a shorter band of 246 bp corresponds to an abnormal transcript without exon 16, whereas the other band of 374 bp corresponds to a normal transcript that contains exon 16. **B**: Ex vivo splicing assays were performed. Wild-type and mutant constructs were stably transfected in HeLa cells, and the exon 16 splicing pattern was analyzed by reverse transcription-polymerase chain reaction. Consistent with data presented in A, exon 16 is predominantly included from the wild-type construct (lane 1). The c.1935G>A mutation at the end of exon 16 resulted in massive skipping of the exon (lane 2). Lane 3 refers to negative control and M indicates size marker. UE and DE refer to upstream and downstream exons of the cassette, respectively.

To investigate the possible effect of the mutation on mRNA splicing, we performed an RT–PCR analysis of total lymphocyte RNA obtained from the patient. The control sample displayed an expected band of 346 bp, containing exons 14 to 17. An additional faint band appeared in the control; it corresponded to an mRNA species missing exon 16 (Figure 3). This pattern was consistently encountered in six tested control individuals (data not shown). This observation suggests that exon 16 must be alternatively spliced, at least in lymphocytes. In the patient however, the splicing pattern showed rather a predominant exclusion of the exon (Figure 3).

To know if the altered splicing is due to the unique mutation found in exon 16, we used a transfected minigene approach to address the functional impact of the c.1935G>A mutation on exon 16 splicing. A genomic fragment of 892 bp containing exon 16 and flanking intron 15 and intron 16 sequences was cloned into an efficient splicing cassette. Control and mutated minigene constructs were stably transfected in HeLa cells. RT–PCR analysis performed on total RNA extracted from transfected cells faithfully reproduced the effects observed in vivo (Figure 3). The minigene splicing pattern obtained from the normal construct revealed a major 374-bp fragment, and a minor 246-bp product. The mutated construct also displayed the two different transcripts, with a predominant shorter band of 246 bp and a faint 374-bp fragment. The proportion (%) of mis-spliced transcripts compared to the full-length transcript was 62%. The percentage was measured using the Quantity One software (Bio-Rad, Marnes-La-Coquette, France; Figure 3). Direct sequencing revealed that the 374 bp contains exon 16, whereas the 246-bp PCR product is lacking the exon. These results strongly suggest that the unique c.1935G>A nucleotide change alters MYO7A pre-mRNA splicing and results in a switch from predominant inclusion to predominant skipping of exon 16.

Exon 16 skipping leads to the loss of the HW helix and the third strand of the central seven-stranded β sheet. The HW helix contains the actin-binding motif, and the strand is part of the central β sheet or transducer region, which concerted distortion led to conformational changes of the motor domain. Therefore, deletion of exon 16 would most likely affect the conformation of MYO7A and would result in a functionally altered allele.

## Discussion

The presence of RP is the crucial trait differentiating Usher syndrome from nonsyndromic HL. RP is defined simply as a progressive retinal dystrophy, identifiable by defective dark adaptation or nyctalopia (night blindness), reduction of the peripheral visual field (known as tunnel vision), appearance of “bony spicule” in the retina revealed by fundoscopy, and an abnormal or nonrecordable ERG [23]. Five autosomal recessive nonsyndromic HL (DFNB2) families with *MYO7A* mutations were reported. In the Chinese, Iranian, and Pakistani families, the affected adults (>25 years of age) who had ophthalmologic examinations were almost normal [10,13,24]. Only one deaf patient (41 years old) in the Pakistani DFNB2 family had slightly subnormal rod and cone responses and a suboptimal quality ERG. However, RP in the Iranian DFNB2 family was ruled out by funduscopy excluding severe but not mild RP. The Tunisian family that enabled the defining of the DFNB2 locus was diagnosed with HL and vestibular dysfunction in 1994, but when reassessed 7 years later, five of 12 adult patients (>25 years of age) were found to have mild RP [25]. The clinical presentation of the Tunisian family we describe in this report is noteworthy because of its similarity to the unrelated Tunisian DFNB2 family reported earlier [25].

To date, over 130 recessive mutations in *MYO7A* have been identified and are listed in the Human Gene Mutation Database. Almost all known *MYO7A* mutations cause USH1B. Six variations were described in five families with autosomal recessive nonsyndromic HL. In the Pakistani DFNB2 family, a correlation of clinical phenotype with the molecular phenotype was observed based on a myosin VIIa intracellular targeting assay. Riazuddin et al. [13] showed that the p.E1716del mutation of myosin VIIa has residual function in the inner ear. However, variations identified in the Chinese and the Iranian DFNB2 families did not document a relationship between the type and location of the *MYO7A* mutations in USH1B and recessive nonsyndromic phenotype. In the Chinese family designated DFNB.05, affected individuals are compound heterozygotes for a splice acceptor consensus site mutation (c.19–2A4G) in intron 3, and a T insertion mutation in exon 28 (p.V1199fsX1228) of *MYO7A* [24,25]. Both of these mutations are predicted to result in premature translation termination, and to date all such predicted truncating alleles of *MYO7A* gene are associated with USH. The p.R244 residue, affected in the Chinese family DFNB.01, is located near the open edge of the large cleft separating the upper 50 kDa from the lower 50K domain [26]. The cleft is thought to close upon binding to actin and open upon dissociation. Introduction of a proline would alter the ability of the cleft to open and close properly upon binding to actin. Proline would also disrupt the hydrogen bond between p.R244 and p.D396 amino acids. The GFP-tagged mouse ortholog of p.R244P (GFP-myosin VIIa [p.R233P]) showed little or no GFP fluorescence within stereocilia, as observed for mutations associated with USH1 [13]. The p.R395H mutation described in the Iranian DFNB2 family affects a residue immediately adjacent to the p.D396. The p.R395 residue, located in the motor domain of myosin VIIa, is exposed and highly conserved across species. The p.R395H mutation induced a local change in charge that likely compromises the structure and/or function of the motor domain. The p.R395 and p.D396 residues are adjacent to p.A397. Watanabe et al. [27] showed that the p.A397D missense mutation associated with USH abolished the actin-activated ATPase activity completely.

In the Tunisian DFNB2 families, the mutation lies at the last nucleotide of exon 16 of the *MYO7A* gene. Modeling analysis has led us to predict that the resultant amino acid change does not impair the structure and the activity of the protein. RNA analyses revealed that exon 16 is slightly excluded in control lymphocytes and that the c.1935G>A mutation triggers enhanced exon skipping. This finding is consistent with our hypothesis that c.1935G>A causes a less severe molecular phenotype than the USH1B-associated alleles. In a mice model Schwander et al. [28] showed that the (c.5742+5G>A) mutation, which affects splicing of the *MYO7A* transcript and truncates the myosin VIIa tail domain, leads to tissue-specific effects on protein levels. In the inner ear, expression of truncated myosin VIIa is severely reduced, whereas in RPE cells, the truncated myosin VIIa is expressed at levels similar to the wild-type protein level.

Previous works have reported four *MYO7A* variants localized at the last position of an exon affecting the G nucleotide at position −1 of donor splice sites. Thus c.592G>A (p.Ala198Thr), c.1687G>A (p.G563S), (c.3503G>C (p.Arg1168Pro), and c.5944G>A (p.Gly1982Arg) were described in families with USH1B. All these sequence variations were shown to globally weaken the natural 5′ splice site and to induce aberrant splicing. Transcripts resulting from the c.1687G>A mutation were undetectable [29]. Minigene studies revealed that c.592G/A, c.3503G/C, and c.5944G/A variants are associated with moderate to high levels of exon skipping [30]. The percentages of mis-spliced transcripts compared to the full-length transcript were respectively 76, 62, and 100%. Although exon skipping is partial in c.592G/A and c.3503G/C mutations and comparable to what we described in Tunisian families, they result in a more severe phenotype. This can be explained by the fact that they occur in compound heterozygotes with a missense p.G163R and a nonsense p.R150X mutation, respectively. These mutations also result in out-of-frame splice products, which would elicit the altered mRNA to degradation by Nonsense-Mediated mRNA Decay (NMD) [31], whereas the c.1935G>A mutation results in in-frame transcript.

In this study, we established that the c.1935G>A mutation identified in Tunisian families with HL and variable expression of RP results in the partial skipping of exon 16. We conclude that the residual function of myosin VIIa may result in DFNB or Usher phenotypes. Other environmental and/or genetic factors might have an impact on the RP phenotype. In this regard, subtle variations in splicing regulation have been recently emphasized regarding the use of the 5′ splice sites [32,33].
